# Amino Acids Drive the Deterministic Assembly Process of Fungal Community and Affect the Flavor Metabolites in *Baijiu* Fermentation

**DOI:** 10.1128/spectrum.02640-22

**Published:** 2023-03-21

**Authors:** Junlin Wei, Jun Lu, Yao Nie, Changwen Li, Hai Du, Yan Xu

**Affiliations:** a Laboratory of Brewing Microbiology and Applied Enzymology, Key Laboratory of Industrial Biotechnology of Ministry of Education, School of Biotechnology, Jiangnan University, Wuxi, China; b Guizhou Guotai Liquor Group Co. Ltd., Guizhou, China; University of Minnesota Twin Cities

**Keywords:** *Baijiu*, amino acid, microbial community assembly, flavor metabolites, metatranscriptomics

## Abstract

Nutrient fluctuation is ubiquitous in fermentation ecosystems. However, the microbial community assembly mechanism and metabolic characteristics in response to nutrient variation are still unclear. Here, we used *Baijiu* fermentation as a case example to study the responses of microbial community assembly and metabolic characteristics to the variation of amino acids using high-throughput sequencing and metatranscriptomics analyses. We chose two fermentation groups (group A with low amino acid and group B with high amino acid contents). The two groups showed similar succession patterns in the bacterial community, whereas they showed different succession in the fungal community wherein *Pichia* was dominant in group A and Zygosaccharomyces was dominant in group B. The β-nearest taxon index (βNTI) revealed that bacterial community was randomly formed, whereas fungal community assembly was a deterministic process. Variance partitioning analysis and redundancy analysis revealed that amino acids showed the largest contribution to the fungal community (37.64%, *P = *0.005) and were more tightly associated with it in group B. Further study revealed that serine was positively related to Zygosaccharomyces and promoted its growth and ethanol production. Metatranscriptomic analysis revealed that the differential metabolic pathways between the two groups mainly included carbohydrate metabolism and amino acid metabolism, which explained the differences of ethanol production and volatile metabolites (such as isoamylol, isobutanol, and 2-methyl-1-butanol). Then these metabolic pathways were constructed and related gene expression and active microorganisms were listed. Our study provides a systematical understanding of the roles of amino acids in both ecological maintenance and flavor metabolism in fermentation ecosystems.

**IMPORTANCE** In spontaneous fermented foods production, nutrient fluctuation is a critical factor affecting microbial community assembly and metabolic function. Revealing the microbial community assembly mechanism and how it regulates its metabolic characteristics in response to nutrient variation is helpful to the management of the fermentation process. This study provides a systematical understanding of the effect of amino acids on the microbial community assembly and flavor metabolisms using *Baijiu* fermentation as a case example. The data of this study highlight the importance of the nutrient management in fermentation ecosystems.

## INTRODUCTION

Traditional fermented foods are produced by naturally inoculated microorganisms through enzymatic conversion and substrate fermentation from raw materials. Microbial community composition and metabolic activities determine the yield and quality of products (1, 2). Recently, our knowledge of the composition and dynamics of complex microbial communities in various fermentation systems has greatly improved with the rapid development of high-throughput sequencing technology (1, 3). However, the assembly patterns of microbial communities in response to a variety of fluctuations in the surrounding environment remain unclear. Nutrient fluctuation is one of the most critical factors affecting microbial community assembly. For example, in the gut microbiome, microbial community composition depends strongly on the diversity of available nutrients (4, 5). Most of the traditional fermented foods are produced using cereals as raw materials, such as *Baijiu* and vinegar (6, 7). The type and hydrolysis degree of raw materials would lead to variations in the content and composition of available nutrients in fermentation systems, thereby affecting microbial growth and metabolism (8, 9). Therefore, revealing the assembly mechanism of microbial communities in response to nutrient variations will not only reveal their ecological mechanism behind form and assembly in fermentation systems but also provide rational strategies to achieve stable production.

Amino acids are very important parameters in fermentation ecosystems. Many studies have found that amino acids act at many levels. First, amino acids are the main nutrients assimilated by microorganisms, after carbon compounds. The growth and fermentation kinetics of microorganisms greatly depend on both the quantity and quality of amino acids (10–12). Second, some amino acids are the precursors for the synthesis of volatile compounds that significantly contribute to the flavor and aroma of products. For example, leucine biosynthesis is highly involved in the formation of isoamylol and its acetate (13–16). In addition, some “functional amino acids,” such as proline, arginine, leucine, valine, cysteine, and methionine, have regulatory roles on yeasts by themselves or cross talk with various metabolic pathways, products, and signal molecules (17). For example, methionine plays an important role as an intracellular methylation agent through the derivative *S*-adenosyl-l-methionine (SAM) (17, 18). Therefore, the management of amino acids in terms of both concentration and composition is fundamental to ensure a steady and successful fermentation. However, most of these studies are performed in experiment conditions by pure cultures, and we still lack knowledge on how amino acids affect microbial community assembly and their metabolic properties in natural fermentation ecosystems.

In this regard, the *Jiang*-flavor *Baijiu* fermentation system represents a typical example of the challenges encountered by microbial communities when it comes to the fluctuation of amino acids. *Jiang*-flavor *Baijiu* is produced by traditional spontaneous solid-state fermentation using sorghum as raw material. The fermentation process is as follows: sorghum is crushed, mixed with water, and then cooked. The cooked grain is cooled and mixed with 10% of *Daqu* powder (starter). This mixture is fermented on the ground (heap fermentation) for 3 days and then moved to a pit and fermented for 30 days (pit fermentation) (Fig. S1 in the supplemental material). During this process, cereal-derived macromolecules are hydrolyzed by multiple enzymes from *Daqu* into available nutrients, which are then converted by yeasts into ethanol and various flavor compounds (19–21). Moreover, *Jiang*-flavor *Baijiu* is produced by repeated batch fermentation and the entire process takes almost a year to complete. During this specific fermentation process, fermented grains are recycled in each batch and production practice has found that nutrient substrates (especially amino acids) are highly variable in each batch. In addition, the yield and quality of *Baijiu* fluctuate in each batch.

In this study, we chose two groups of *Jiang*-flavor *Baijiu* fermentation with different contents of amino acids. The effect of amino acids on microbial community assembly and metabolic function was identified using and integrating omics techniques (high-throughput sequencing and metatranscriptomics) with measured fermentation parameters and flavor metabolites.

## RESULTS

### Dynamics of fermentation parameters and volatile metabolites during *Baijiu* fermentation.

Group A had a lower content of amino acids than that in group B. At the start of fermentation (H1), the content of total amino acids was 5.55 ± 0.10 g/kg in group A, which was significantly lower (*P < *0.01) than that in group B (7.79 ± 0.07 g/kg) (Table 1). Moreover, group A had lower contents of total amino acids than group B during both heap and pit fermentation (Fig. S2). In detail, the contents of 17 free amino acids in two groups are shown in Table S1. Further analysis revealed that serine, valine, and methionine showed significant differences (*P < *0.05) between the two groups, and they all had higher contents in group B. Serine content was 90.01 ± 9.46 mg/kg in group A at H1, whereas it was 335.78 ± 22.75 mg/kg in group B. Valine content was 286.78 ± 10.64 mg/kg in group A at H1, whereas it was 378.65 ± 5.97 mg/kg in group B.

**TABLE 1:** Contents of total amino acids in group A and group B during fermentation^*a*^

Fermentation days	Group A	Group B
H1	5.55 ± 0.10	7.79 ± 0.07^*b*^
H2	5.24 ± 0.07	6.67 ± 0.01^*b*^
H3	5.72 ± 0.03	5.92 ± 0.06
F00	5.76 ± 0.04	5.97 ± 0.10
F05	5.73 ± 0.10	5.60 ± 0.09
F10	5.69 ± 0.11	5.82 ± 0.03
F15	5.68 ± 0.07	5.87 ± 0.05
F30	5.79 ± 0.06	6.09 ± 0.03

a Values are in g/kg; *n* = 4. H, heap fermentation. F, pit fermentation.

b Adjusted *P < *0.05 (Tukey’s test).

**FIG 1:**
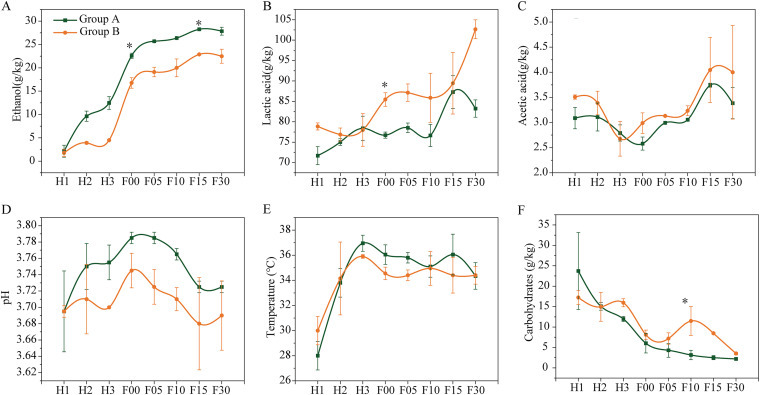
Dynamics of the fermentation parameters in two groups. (A) Ethanol. (B) Lactic acid. (C) Acetic acid. (D) pH. (E) Temperature. (F) Carbohydrates. Each value represents the average and error bars represent the standard deviation (*n* = 4). *, Adjusted *P < *0.05 (Tukey’s test). H, heap fermentation; F, pit fermentation. The numbers after the letters represent the number of fermentation days.

Fermentation parameters of the two groups were highly different (Fig. 1 and Fig. S3). Group A had a higher level of ethanol than group B during both heap and pit fermentation (Fig. S3A). At the start of heap fermentation, the ethanol contents in groups A and B were equivalent. At the start of pit fermentation (F00), the ethanol content showed a significant (*P < *0.05) difference between the two groups and it was 22.58 ± 0.55 g/kg in group A and 16.76 ± 1.13 g/kg in group B (Fig. 1A). Lactic acid content was significantly (*P < *0.01) higher in group B during the pit fermentation (Fig. S3B), mainly at the start of pit fermentation (F00) (Fig. 1B). Lactic acid content reached 85.46 ± 1.65 g/kg at the start of pit fermentation in group B, whereas it was 76.73 ± 0.75 g/kg in group A. Acetic acid showed no significant difference between two groups (Fig. 1C and Fig. S3C). Although temperature and pH showed no significant difference between two groups in each fermentation time, they were significantly higher in group A during pit fermentation (Fig. S3D and E). Carbohydrates were significantly higher in group B at the pit fermentation, mainly at F10 (Fig. 1F and S3F).

**FIG 2:**
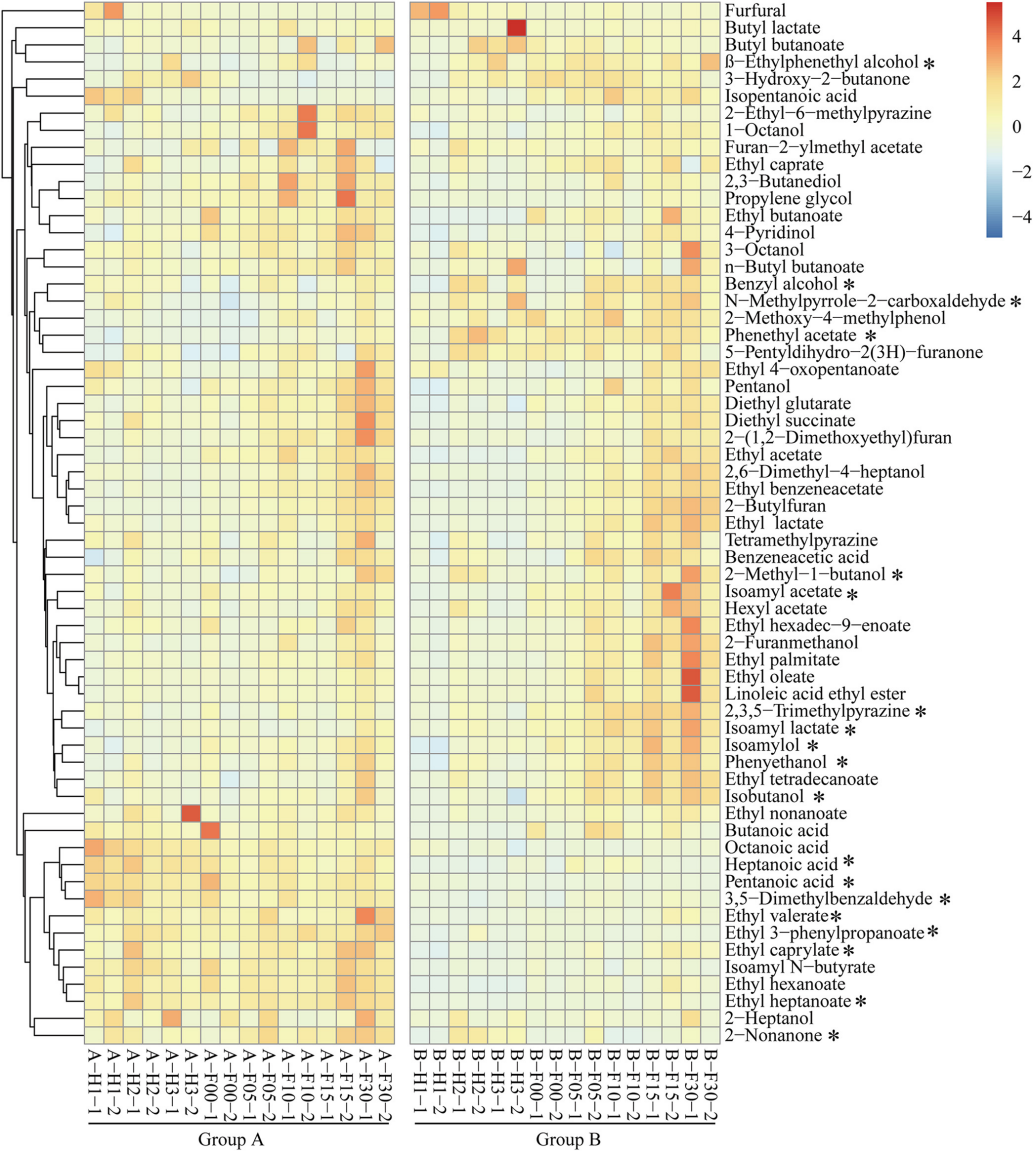
Heatmap of the volatile metabolites in two groups during fermentation. The color scale represents the scaled abundance of each metabolite, indicated as the *Z*-score, with red and blue indicating high and low abundances, respectively. H, heap fermentation; F, pit fermentation. The numbers after the letters represent the number of fermentation days; “−1” represents one batch in a group and was averaged by two sample points (see sample collection); and “−2” represents another batch in a group. *, Adjusted *P < *0.05 (Tukey’s test).

A total of 62 volatile metabolites (28 esters, 14 alcohols, 5 organic acids, 9 aromatics, and 2 other compounds) were identified from all the samples (Fig. 2 and Fig. S4). Nineteen volatile metabolites were significantly different between the two groups. Among them, β-ethylphenethy alcohol, benzyl alcohol, *N*-methylpyrrole-2-carboxaldehyde, phenethyl acetate, 2-methyl-1-butanol, isoamyl acetate, 2,3,5-trimethylpyrazine, isoamyl lactate, isoamylol, phenyethanol, and isobutanol showed significantly (*P < *0.05) higher contents in group B than group A. However, the contents of heptanoic acid, pentanoic acid, 3,5-dimethylbenzaldehyde, ethyl valerate, ethyl-3-phenylpropanoate, ethyl caprylate, ethyl heptanoate, and 2-nonanone were significantly (*P < *0.05) higher in group A.

### Microbial diversity and composition during *Baijiu* fermentation.

High-throughput sequencing was used to characterize microbial diversity and community structures during *Baijiu* fermentation. After quality control, 1,701,325 high-quality reads from the internal transcribed spacer (ITS) region and 1,264,760 high-quality reads from the V3 to V4 region of the 16S rRNA gene were obtained from all the samples. All Good’s coverage values exceeded 99.80%, indicating that the majority of the microbiota was represented by the sequences.

**FIG 3:**
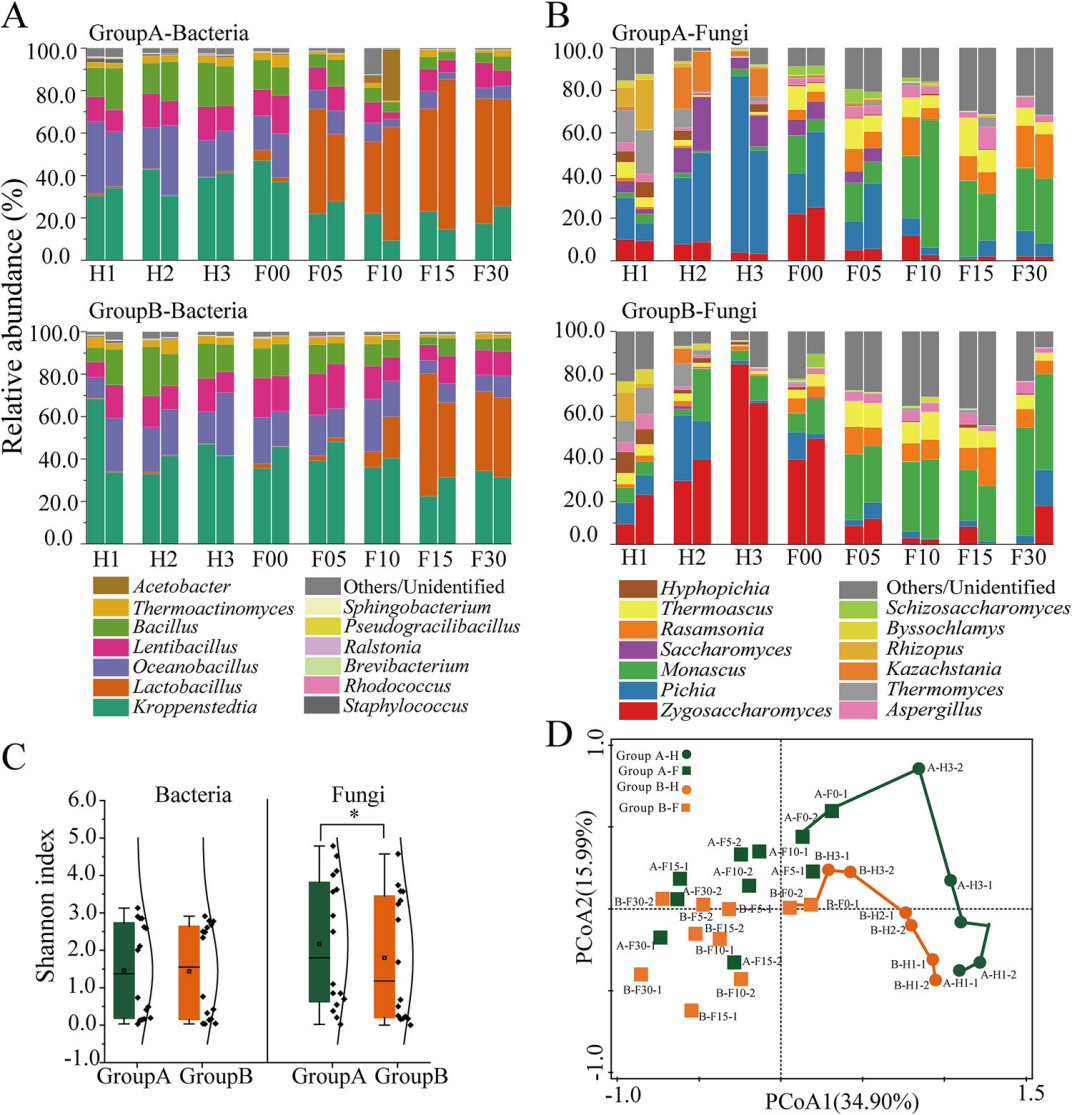
Microbial communities during *Baijiu* fermentation. Abundance and succession of bacterial communities (A) and fungal communities (B) in two groups at the genus level. Only genera that had an average abundance of > 1% are indicated. Each bar represents one batch in a group, and was averaged by two sample points. (C) Microbial community diversity based on the Shannon index. *, *P < *0.05, Tukey’s test. (D) Principal-component analysis (PCoA) based on the Bray-Curtis dissimilarity matrix for microbial communities. H, heap fermentation; F, pit fermentation. The numbers after the letters represent the number of fermentation days; “−1” represents one batch in a group and was averaged by two sample points; and “−2” represents another batch in a group.

Microbial alpha diversity was calculated based on the Shannon index. Fungal diversity was significantly higher (*P < *0.05) in group A than that in group B (Fig. 3C). Principal-component analysis (PCoA) based on the Bray-Curtis distance matrix at the operational taxonomic unit (OTU) level indicates that the microbial communities of the two groups were distinctly separate at the early stage of fermentation (H1, H2, H3, and F00) and were similar after the fifth days of pit fermentation (Fig. 3D).

To illustrate the significant contributors of the two groups, the relative abundance of the microorganisms was determined at the genus level. At the heap fermentation, the largest groups of bacterial genera were *Kroppenstedtia*, *Oceanobacillus*, *Lentibacillus*, and *Bacillus*, whereas *Lactobacillus* was gradually dominant at the pit fermentation. *Lactobacillus* showed a different succession rate between groups A and B. *Lactobacillus* became the dominant genus at F05 in group A, whereas it was at F10 in group B. The relative abundance of *Lactobacillus* in group A was always higher than that in group B at the pit fermentation (Fig. 3A).

At the start of heap fermentation, the fungal genus included yeasts and filamentous fungi. However, yeasts quickly dominated and *Pichia* dominated in group A and Zygosaccharomyces dominated in group B (Fig. 3B). Two genera reached their maximum levels at the end of the heap fermentation (H3) when the abundance of *Pichia* was 65.71 ± 17.12% in group A and Zygosaccharomyces was 75.55 ± 13.33% in group B. Then, filamentous fungi were gradually dominant in both two groups, including *Monascus*, *Rasamsonia*, and *Thermoascus*, among which *Monascus* showed the maximum abundance.

The cooccurrence networks of the microbial communities indicated that microbial interactions were different between two groups (Fig. S5). First, two network indices, modularity and average degree were estimated to assess the topological properties of microbial networks (22). Group B (0.457) had greater modularity than group A (0.219), suggesting that the microbial community in group B was more resilient to environmental stresses during fermentation as different species had better division and can complement each other (23). The average degree means the connectivity, revealing the strength of a species, is connected to other species. A higher average degree in group A revealed that microorganisms in the network possessed a large number of connections (22). Moreover, the positive relationship between microorganisms in group B was higher than that in group A. Zygosaccharomyces had more negative relationships with other genera in group B, such as *Rasamsonia*, *Thermoascus*, Aspergillus, *Penicillium*, and *Lactobacillus*, and had a positive relation with *Kazachstania* and *Bacillus*, whereas Zygosaccharomyces was only positively related to *Schizosaccharomyces* in group A. *Pichia* had 7 connections in group A, whereas had no connections in group B. *Lactobacillus* had stronger connections with bacterial genera in group A, whereas it was only negatively related to *Bacillus* in group B. These results revealed that amino acids might have significant effects on the microbial interaction.

**FIG 4:**
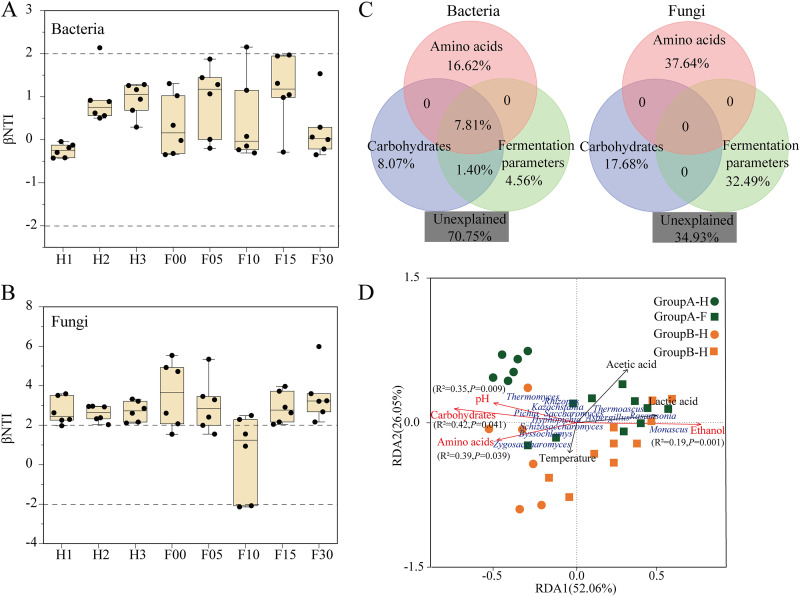
Ecological processes and significant environmental factors driving microbial community assembly during *Baijiu* fermentation. (A) βNTI values of bacterial community during fermentation. (B) βNTI values of fungal community during fermentation. (C) Variation partitioning analysis (VPA) shows the contribution of amino acids, carbohydrates and fermentation parameters to the microbial communities. (D) Redundancy analysis (RDA) plots shows the weights and orientation of fungal communities and environmental parameters. H, heap fermentation; F, pit fermentation. The numbers after the letters represent the number of fermentation days; “−1” represents one batch in a group and was averaged by two sample points.

Microbial community assembly patterns and determinants during *Baijiu* fermentation. β-Nearest taxon index (βNTI) values were determined across all pairwise community comparisons in each fermentation time between two groups to determine the ecological processes affecting microbial community assembly. For bacteria, all the |βNTI| values were between −2 and 2, revealing that the bacterial community was dominated by a stochastic process (Fig. 4A). For fungi, the βNTI values between different samples were >2, indicating that the deterministic process was important for shaping the fungal community; here, variable selection played a key role (Fig. 4B). Exceptionally, −2 < βNTI < 2 at F10 revealed a stochastic assembly for fungi community.

Furthermore, we investigated the main drivers of microbial community assembly by variance partitioning analysis (VPA) and redundancy analysis (RDA) analysis. Considering the overwhelming number of environmental variables in the samples, we divided environmental factors into three groups for VPA analysis, including carbohydrates, amino acids, and fermentation parameters (pH, temperature, lactic acid, ethanol, and acetic acid). For the bacterial community, these factors together explained 29.25% of the observed variation, leaving 70.75% of the variation unexplained. Amino acids explained 16.62% of the variation, followed by carbohydrates (8.07%) and fermentation parameters (4.56%). For the fungal community, these factors together explained 65.07% of the observed variation, leaving 34.93% of the variation unexplained. Amino acids explained the largest portion (37.64%, *P = *0.005) of the observed variation, followed by fermentation parameters (32.49%, *P = *0.002) and carbohydrates (17.68%) (Fig. 4C). These results revealed that amino acids significantly contributed to the fungal community assembly during *Baijiu* fermentation.

RDA analysis was used to systematically clarify the driving force of the fungal community in two groups at different fermentation stages. The two axes contributed to 78.11% of the total variance in fungal communities, indicating the remarkable correlations between fungal communities and these parameters. At the heap fermentation, pH (*R*^2^ = 0.35, *P = *0.009), carbohydrates (*R*^2^ = 0.42, *P = *0.041), and amino acids (*R*^2^ = 0.39, *P = *0.039) were greatly associated with fungal communities. In addition, amino acids were more tightly related to the fungal community in group B. However, ethanol (*R*^2^ = 0.19, *P = *0.001) was the main driver of the fungal community at the pit fermentation (Fig. 4D).

### Microbial metabolic function analysis by metatranscriptomic.

**FIG 5:**
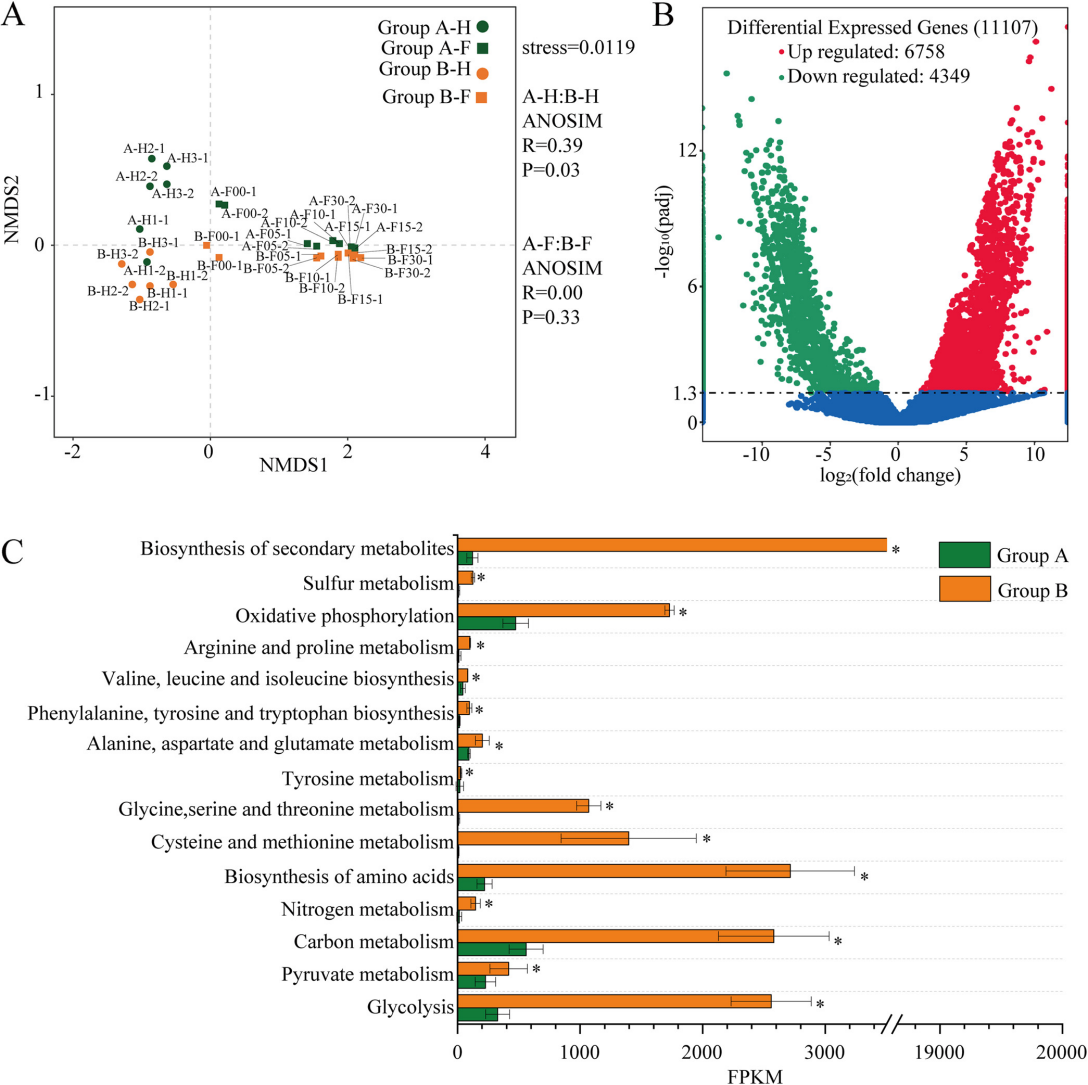
Metabolic function difference analysis between the two groups by metatranscriptomic. (A) Nonmetric multidimensional scaling (NMDS) plot of the samples based on the gene expressionH, heap fermentation; F, pit fermentation. The numbers after the letters represent the number of fermentation days; “−1” represents one batch in a group and was averaged by two sample points. (B) Volcano plot of the numbers of differentially expressed genes between the two groups. The red represents the upregulated and the green represents the downregulated genes in group A. The blue represents that these genes had no differential expression between the two groups. (C) Kyoto Encyclopedia of Genes and Genomes (KEGG) pathways of differentially expressed genes and their expression levels indicated by fragments per kilobase of transcript per million mapped reads (FPKM). *, Adjusted *P < *0.05, Tukey’s test.

To reveal the metabolic differences of microbial communities between the two groups, the gene expression patterns were analyzed by metatranscriptomic analysis. Nonmetric multidimensional scaling (NMDS) showed two distinct clusters referring to group A and group B at the heap fermentation (Fig. 5A; Anosim test, *R* = 0.39, *P = *0.03). However, no distinct cluster was found between the two groups at the pit fermentation (*R* = 0.00, *P = *0.33). These results revealed that the metabolic activities of microbial communities between groups A and B had a greater difference at the heap fermentation but tended to be consistent at the pit fermentation. Furthermore, 11,107 genes were found to be significantly differentially expressed between two groups, including 6,758 upregulated genes and 4,349 downregulated genes in group A at the heap fermentation, compared to group B.

Based on the KEGG pathway annotation, these differential genes were found to be mainly distributed in the glycolysis, pyruvate metabolism, carbon metabolism, nitrogen metabolism, biosynthesis of amino acids, cysteine and methionine metabolism, glycine, serine and threonine metabolism, tyrosine metabolism, alanine, aspartate, and glutamate metabolism, phenylalanine, tyrosine and tryptophan biosynthesis, valine, leucine, and isoleucine biosynthesis, arginine and proline metabolism, oxidative phosphorylation, sulfur metabolism and biosynthesis of secondary metabolites (Fig. 5C). The gene expression values of these pathways were higher in group B than those in group A.

**FIG 6:**
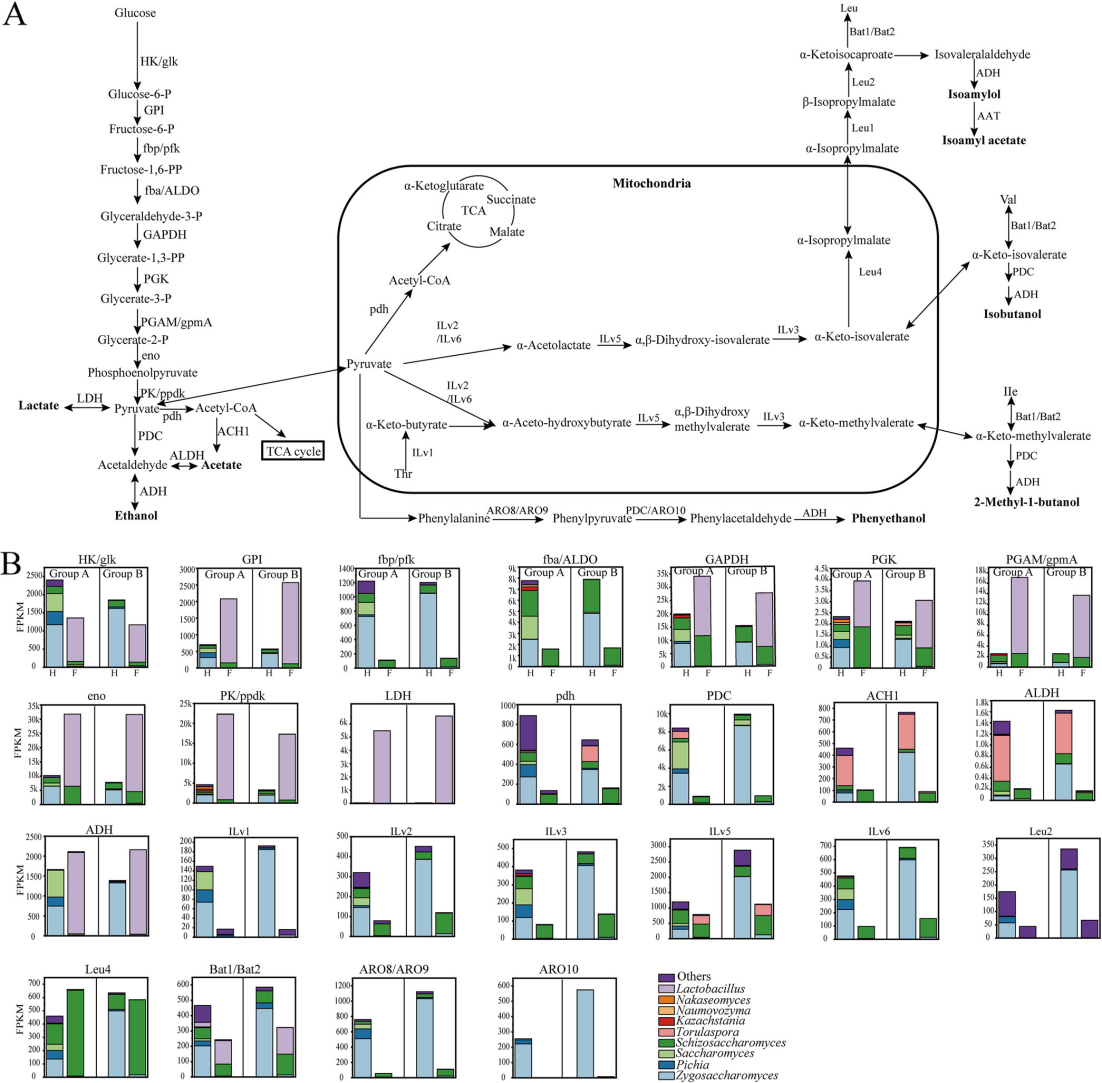
Metabolic pathways of ethanol, lactate, and acetate production as well as flavor formation. The related enzymes and the contributed microorganisms are listed. (A) Metabolic pathways. (B) The expression levels of the genes encoding related enzymes in the two groups. H represents heap fermentation and F represents pit fermentation. Each bar presents the average value at the heap/pit fermentation. HK, hexokinase (K00844); glk, glucokinase (K00845); GPI, glucose-6-phosphate isomerase (K01810); fbp, fructose-1,6-bisphosphatase I (K03841); pfk, 6-phosphofructokinase 1 (K00850); fba, fructose-bisphosphate aldolase, class II (K01624); ALDO, fructose-bisphosphate aldolase, class I (K01623); GAPDH, glyceraldehyde 3-phosphate dehydrogenase (phosphorylating) (K00134); PGK, phosphoglycerate kinase (K00927); PGAM, 2,3-bisphosphoglycerate-dependent phosphoglycerate mutase (K01834); gpmA, 2,3-bisphosphoglycerate-dependent phosphoglycerate mutase (K15634); eno, enolase (K01689); PK, pyruvate kinase (K00873); ppdk, pyruvate, orthophosphate dikinase (K01006); LDH, l-lactate dehydrogenase (K00016); pdh, pyruvate dehydrogenase E1 component alpha subunit (K00161); PDC, pyruvate decarboxylase (K01568); ALDH, aldehyde dehydrogenase (NAD+) (K00128); ADH, alcohol dehydrogenase (K13951); ACH1, acetyl-CoA hydrolase (K01067); Ilv1, threonine deaminase (K01754); Ilv2, acetolactate synthase I/II/III large subunit (K01652); Ilv3, dihydroxyacid dehydratase (K01687); Ilv5, ketol-acid reductoisomerase (K00053); Ilv6, acetolactate synthase I/III small subunit (K01653); Leu4, 2-isopropylmalate synthase (K01649); Leu1, isopropylmalate isomerase (K01704); Leu2, 3-isopropylmalate dehydrogenase (K00052); Bat1, branched-chain amino acid aminotransferase (K00826); Bat2, cytosolic BCAA aminotransferase; DC, α-keto acid decarboxylase (K01568); AAT, alcohol aminotransferase (K13953); KDC, α-keto acid (pyruvate) decarboxylase (K01568); ARO8, aromatic amino acid aminotransferase I/2-aminoadipate transaminase (K00838); ARO9, aromatic amino acid aminotransferase II (K05821); ARO10, phenylpyruvate decarboxykase (K12732).

The above results showed that amino acid metabolism and carbohydrate metabolism explained the largest differences in microbial metabolic activities between the two groups. Amino acid metabolism and carbohydrate metabolism are highly involved in the higher alcohol formation and ethanol production, which may explain the differences in higher alcohol and ethanol between the two groups. Thus, we constructed the related metabolic pathways and analyzed the gene expression levels, as shown in Fig. 6. Active microorganisms that participated in these metabolic pathways were fungal members, including Zygosaccharomyces, *Pichia*, *Saccharomyces*, *Schizosaccharomyces*, *Torulaspore*, *Kazachstania*, *Naumovozyma*, and *Nakaseomyces*, and bacterial member *Lactobacillus*.

For glycolysis, the genes encoding hexokinase, glucose-6-phosphate isomerase, fructose-1,6-bisphosphatase, fructose-bisphosphate aldolase, glyceraldehyde 3-phosphate dehydrogenase, phosphoglycerate kinase, 2,3-bisphosphoglycerate-dependent phosphoglycerate mutase, enolase, and pyruvate kinase were mainly expressed by fungal members at the heap fermentation and were expressed by *Lactobacillus* at the pit fermentation. In pyruvate metabolism, the gene encoding lactate dehydrogenase (LDH) was mainly expressed by *Lactobacillus* at the pit fermentation, and the expression level was higher in group B. The gene encoding alcohol dehydrogenase (ADH) was expressed by Zygosaccharomyces, *Pichia*, and *Saccharomyces* in group A and was only expressed by Zygosaccharomyces in group B at the heap fermentation, whereas it was expressed by *Lactobacillus* at the pit fermentation in both two groups. In addition, the expression level of ADH was higher in group B than that in group A. These results were consistent with a higher ethanol yield and lower lactate in group A.

Leucine, isoleucine, and valine biosynthesis were highly involved in the formation of isoamylol, isoamyl alcohol, isobutanol, and 2-methyl-1-butanol. Isoamylol is mainly converted from α-ketoisocaproate, an intermediate of the leucine synthetic pathway from glucose, in two steps catalyzed by α-keto acid decarboxylase and ADH. The gene encoding α-keto acid decarboxylase had a higher expression level in group B and was mainly produced by Zygosaccharomyces at the heap fermentation and by *Lactobacillus* at the pit fermentation. Valine biosynthesis was highly related to isobutanol formation by key intermediate α-ketoisovalerate. α-Ketoisovalerate was then translated into isobutanol by pyruvate decarboxylase (PDC) and ADH. PDC had a higher expression level in group B and was mainly produced by Zygosaccharomyces. Isoleucine is involved in the synthesis of 2-methyl-1-butanol by pyruvate decarboxylase and alcohol dehydrogenase. Phenylalanine was involved in the formation of phenylethanol by aromatic amino acid aminotransferase, phenylpyruvate decarboxylase, and alcohol dehydrogenase. The genes encoding these enzymes had higher expression in group B and were mainly produced by Zygosaccharomyces. These results were consistent with the higher contents of isoamylol, isoamyl alcohol, isobutanol, 2-methyl-1-butanol, and phenylethanol in group B. Therefore, amino acids play a critical role in flavor formation. Meanwhile, we found that Zygosaccharomyces was a critically active microorganism contributing to amino acid metabolism and flavor production during *Baijiu* fermentation.

### Verification of the effects of amino acids on the growth and fermentation performance of yeasts.

**FIG 7:**
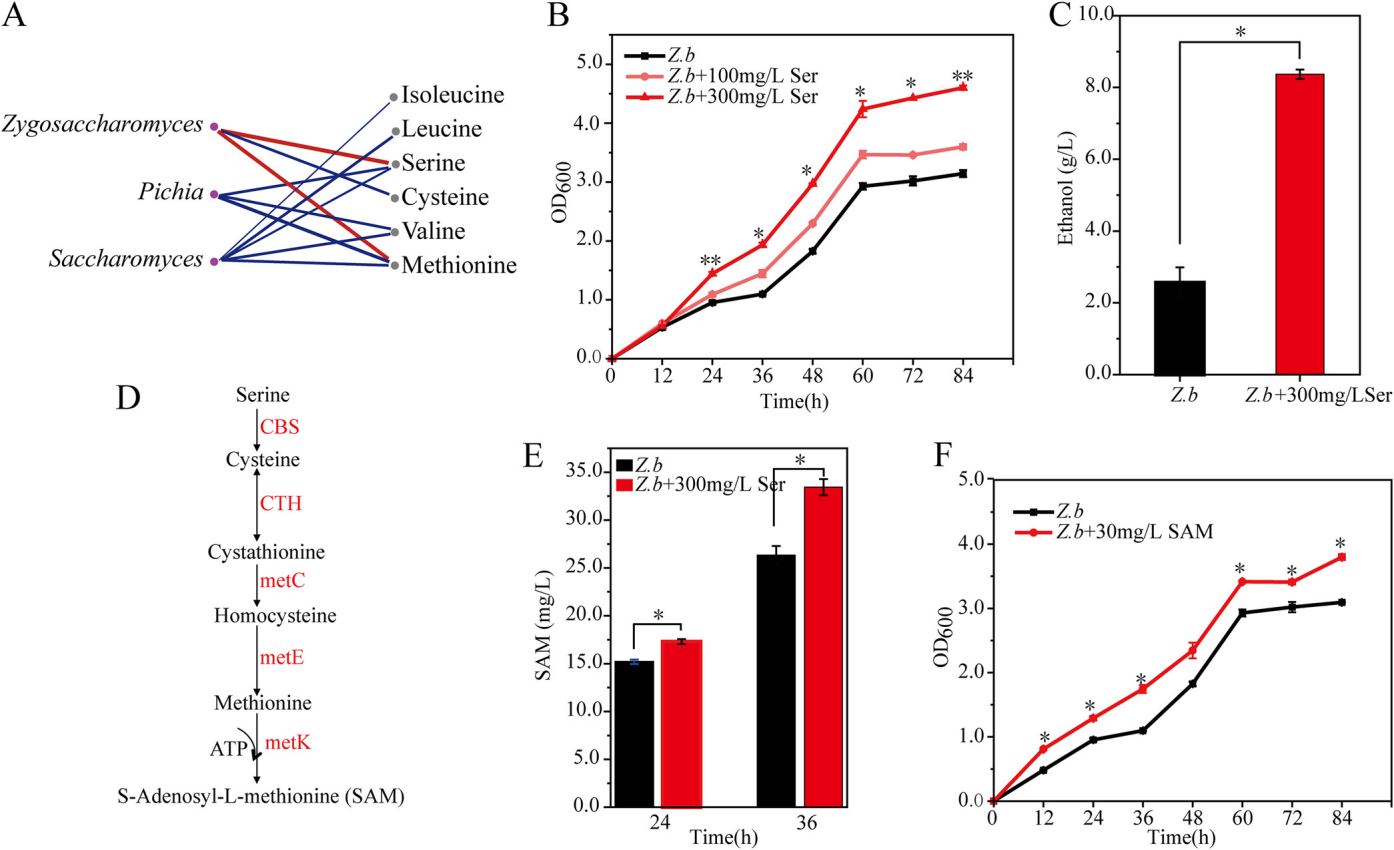
The effect of amino acids on the growth and fermentation performance of yeasts. (A) The relationships between amino acids and yeasts. A connection indicates a significant (adjusted *P < *0.05) and strong (Spearman’s |ρ| > 0.6) correlation. The thickness of each connection (edge) between two nodes is proportional to Spearman’s correlation coefficient (ρ). The color of each edge corresponds to a positive (red) or negative (blue) relationship. (B) OD_600_ values of Z. bailii during fermentation in the presence of 100 mg/L and 300 mg/L serine (*n* = 3). (C) The ethanol production by Z. bailii at the end of fermentation (96 h) in the presence of 300 mg/L serine (*n* = 3). (D) The metabolic pathway from serine to S-Adenosyl-l-methionine. The genes encoding enzymes are indicated in red. CBS, cystathionine beta-synthase; CTH, cystathionine gamma-lyase; metC, cysteine-S-conjugate beta-lyase; metE, 5 methyltetrahydropteroyltriglutamate–homocysteine methyltransferase; metK, S-adenosylmethionine synthetase. (E) The SAM content produced by Z. bailii at 24 h and 36 h of fermentation in the presence of 300 mg/L serine (*n* = 3). (F) OD_600_ values of Z. bailii during fermentation in the presence of 30 mg/L SAM (*n* = 3). *, Adjusted *P < *0.05, Tukey’s test.

With the results that amino acids were related to the yeast assemble and succession, Spearman correlation analysis was performed to explore the association between key yeasts and specific amino acids. Three yeasts were significantly (|ρ| > 0.60 and *P < *0.05) related to 6 amino acids (Fig. 7A). Only Zygosaccharomyces was positively related to serine and methionine. Moreover, serine and methionine had significantly (*P < *0.05) higher contents in group B than those in group A. We speculated that these two amino acids may promote the dominance of Zygosaccharomyces. Therefore, we explored the effect of serine and methionine on the growth and fermentation performance of Zygosaccharomyces. According to metatranscriptomic analysis, the active species of the Zygosaccharomyces genus is Zygosaccharomyces bailii. Then, the strain Z. bailii was isolated from fermented grains.

Compared to the control group, serine supplementation promoted the growth of Z. bailii; in particular, 300 mg/L serine exhibited the maximum promotion (Fig. 7B), which was in accordance with the previous results. However, no significant promotion (*P > *0.05) was observed from methionine supplementation (Fig. S7). In the presence of 300 mg/L serine, opitcal density at 600 nm (OD_600_) of Z. bailii was 4.60 ± 0.04 at the end of fermentation, which was significantly higher (*P < *0.01) than that in the control group (3.14 ± 0.06). We noticed that 300 mg/L serine supplementation increased the growth of Z. bailii from the 24 h of fermentation. Specific metabolites may be produced during serine metabolism to promote the growth of Z. bailii. Then, SAM was considered a putative metabolite according to previous studies (24, 25). The metabolic pathway from serine to SAM was shown in Fig. 7D. Next, we monitored the transcription levels of related genes by quantitative PCR at 24 h and 36 h of fermentation. The results revealed that serine supplementation increased the transcription levels of these genes, compared to the control (Fig. S8). Correspondingly, the SAM content was significantly (*P < *0.05) increased by serine supplementation at 24 h and 36 h of fermentation (Fig. 7E). At 24 h, the SAM content was 17.31 ± 0.26 mg/L in the serine group, whereas it was 15.19 ± 0.23 mg/L in the control group. At 36 h, the SAM content was 33.43 ± 0.84 mg/L in the serine group, whereas it was 26.33 ± 0.96 mg/L in the control group. Finally, we added the SAM (30 mg/L) to the culture of Z. bailii to verify its promotion. The results revealed that SAM addition significantly increased the growth of Z. bailii (Fig. 7F). More importantly, the presence of 300 mg/L serine significantly (*P < *0.05) increased the ethanol production by Z. bailii at the end of fermentation, which increased from 2.56 ± 0.42 g/L to 8.37 ± 0.13 g/L (Fig. 7C). Therefore, these results indicated that the high content of serine promoted the dominance of Zygosaccharomyces in group B by promoting its growth and ethanol production in the high-acidity *Baijiu* fermentation system.

## DISCUSSION

In spontaneous fermentation ecosystems, microbial communities have self-assembled according to the environmental conditions in which they live. However, environmental fluctuations are ubiquitous because of the spontaneous fermentation of fermented foods. Knowledge of the microbial community assembly mechanism under a variety of fluctuations is essential for the management of fermentation process. In this study, we examined the microbial community assembly and metabolic properties response to the variations of amino acids using *Jiang*-flavor *Baijiu* as a case study. Our results showed that amino acids were the main driver affecting the deterministic assembly process of fungal community, mainly for yeasts. In addition, amino acids were highly involved in the production of flavor compounds related to *Baijiu* quality during fermentation. Our study provides a systematical understanding of the role of amino acids in both ecological maintenance and flavor metabolism during *Baijiu* fermentation.

Bacterial community assembly was governed by a stochastic process, suggesting that bacteria were formed randomly and might not be affected by environmental selection. We speculated that the predominant bacteria originated from *Daqu* and they can quickly adapt to the fermentation environment (26). As a starter, *Daqu* provides abundant microorganisms for *Jiang*-flavor *Bajiu* production. Many researchers have revealed the microbial community structure in *Daqu*. The dominant bacterial genera in *Daqu* are *Bacillus*, *Virgibacillus*, *Kroppenstedtia*, *Oceanobacillus*, and *Lactobacillus*. For fungi, the dominant genera are *Thermomyces*, *Thermoascus*, and Aspergillus, whereas yeasts have very low abundance (27, 28). Therefore, this is consistent with a previous study where authors found that *Daqu* provided 95.6% of bacteria to fermentation grains at the initial heap fermentation in *Jiang*-flavor *Baijiu* fermentation, whereas, fungi, especially yeasts, mainly originate from the fermentation environments, such as indoor ground and tools (29). βNTI values indicated that fungal community assembly was a deterministic process, revealing that the microorganisms from environments were deterministically shaped and were strongly selected by environmental conditions (29). At the start of fermentation, the fungal genera in two groups contained multiple yeasts and filamentous fungi; however, yeasts quickly were dominated at the heap fermentation and filamentous fungi were dominated at the later stage of pit fermentation (Fig. 3B). These results revealed that initial environment selection mainly acted on yeasts, whereas the changes of fermentation parameters induced by microbial metabolism at the early stage, known as niche modification, further mediated the environmental selection on filamentous fungi. Compared with carbohydrates and fermentation parameters, amino acids had the largest contribution to fungal community assembly. Amino acids and carbohydrates were the determinants at the heap fermentation and ethanol was the determinant at the later stage of pit fermentation driving fungal community assembly (Fig. 4D). This is consistent with the characteristics of *Jiang*-flavor *Baijiu* production, where cereal-derived macromolecules are hydrolyzed by multiple enzymes from *Daqu* into available nutrients (such as fermentable sugars and amino acids) used for the energy substrate of yeast growth. Then ethanol production by yeasts gradually increases and mediates the growth of other microorganisms, such as *Lactobacillus* and filamentous fungi (7, 30). This result is consistent with a recent study where microbial community assembly during saccharification is random; however, niche modification induced by saccharification determines the microbial community assembly during the later fermentation process in *Xiaoqu* liquor brewing (31).

*Pichia* was dominant in group A and Zygosaccharomyces was dominant in group B at the heap fermentation (Fig. 3B), suggesting that these two yeasts showed preferences for amino acids for sustaining growth and fermentation. This is consistent with the dynamics of amino acids, which showed a decline throughout the heap fermentation and then gradually increased during the pit fermentation, especially in group B (Table 1). The initial decline of amino acids can be explained by the utilization by yeast cells for their growth and metabolism. The later increase of amino acids may be due to the autophagy and lysis of cells because of the increase of environmental stresses (such as lactic acid and ethanol) during pit fermentation. Among amino acids, serine was positively related to Zygosaccharomyces and promoted its growth (Fig. 7). This result is consistent with previous studies. For example, yeast growth and fermentation properties greatly depend on the quality and nature of available amino acids in the wine industry (11, 12). According to the capacity to support yeast growth and fermentation activity, amino acids can be divided into poor and rich nitrogen sources (32). We demonstrated that serine promoted the growth of Zygosaccharomyces by producing SAM. SAM is the principal methyl donor for methylation reactions, which is central to regulating many biological processes, such as metabolism, signal transduction, and gene expression (33). In addition, recent studies have documented that stimulating SAM synthesis extended the life span of S. cerevisiae by activating AMP-activated protein kinase, as well as increased the tolerance of yeasts to high-maltose conditions (34, 35). Most importantly, we demonstrated that serine significantly promoted ethanol yield by Zygosaccharomyces (Fig. 7C). During the *Baijiu* fermentation process, the pH of fermented grains was lower than 4.0 (Fig. 1D). Survival and production of ethanol in such an acidic environment are a challenge for yeasts. Our results revealed that serine may be necessary for Zygosaccharomyces during *Baijiu* fermentation. Zygosaccharomyces is an important yeast in fermented food production due to its ability to produce flavor compounds and good tolerance toward various environmental stresses. Zygosaccharomyces could produce important flavor compounds in fermented foods, such as isoamylol, pentanol, and isobutanol (36). Most importantly, its strong tolerance to various stresses ensures the ecology and biological activity in fermentations, especially in acidic food products. Previous studies have indicated that Zygosaccharomyces is the most tolerant yeast species to weak acids (37). Its tolerance mechanisms to multiple acids have been revealed by transcriptomic analysis, including the transcriptional activation mediated by transcription factors (such as *ZbMsn4* and *ZbHaa1*) and the modulation of cell membrane permeability and the activity of transporters (38, 39). Interestingly, recent studies revealed that exogenous supplementation of some compounds provided stress protection for Zygosaccharomyces. For example, supplementation of Tartary buckwheat protein hydrolysates and fatty acids improved the salt tolerance of Zygosaccharomyces (40, 41). These results give evidence that some valuable compounds are closely linked to the stress tolerance of microorganisms in fermentation systems. Therefore, our results might provide a new insight that serine is another mechanism of lactic acid tolerance of Zygosaccharomyces during *Baijiu* fermentation. However, the exact mechanism needs further exploration.

Yeasts are responsible for ethanol production and flavor formation during *Baijiu* fermentation. However, the occurrence of different yeasts exhibited different fermentation performances (20, 42). This study provides guidance that amino acid may be a critical factor affecting the structure and diversity of yeasts during *Baijiu* fermentation. In addition, only sorghum was used as the raw material and *Daqu* as the starter for *Jiang*-flavor *Baijiu* production in this study. However, multiple combinations of cereals (rice, sticky rice, sorghum, wheat, and corn) and starter are used to produce other types of *Baijiu*, in which protein contents are highly variable, then resulting in the differences in available amino acids in fermentation systems (7, 43, 44). The availability of amino acids determines the scaling of community properties. Therefore, more attention should focus on the effect of amino acids on the microbial community and design more efficient *Baijiu* fermentation systems.

Microbial communities exert their function properties in response to a variety of possible fluctuations in the surroundings through differential gene expression, further resulting in the change of flavor metabolites in fermented foods. When regarding to the changes of available nutrients, microorganisms usually regulate their metabolic phenotypes by sensing the availability of specific nutrients and then synthesizing the enzymes required for their catabolism (45, 46). Therefore, metatranscriptomic analysis is essential to explore the active microbial members and their expression genes related to ethanol and flavor compounds production during *Baijiu* fermentation. Ethanol is a very important indicator of *Baijiu* fermentation performance (42). Multiple yeasts (*Pichia*, *Saccharomyces*, and Zygosaccharomyces) expressed high levels of alcohol dehydrogenase (ADH) in group A, whereas only Zygosaccharomyces expressed ADH in group B. These results showed that yeast diversity was positively related to ethanol production during *Baijiu* fermentation. The relationship between microbial diversity and community function has been explored in ecosystems. For example, high microbial diversity increased the soil ecosystem function (47–50). Flavor compounds are key components used for evaluating consumer preference and control targets in fermented foods production (51). Significant differences in volatile metabolites were observed between the two groups. Alcohols and their acetate had higher contents in group B, whereas acids (such as butanoic acid, heptanoic acid, and pentanoic acid) had higher contents in group A, revealing that gene expression encoding the enzymes required for their metabolism had differences between the two groups. Higher alcohols, such as isoamylol, isoamyl alcohol, isobutanol, and 2-methyl-1-butanol, are one of the main aroma compounds and contribute to the mellow and sweet taste of *Baijiu* (52, 53). In this study, Zygosaccharomyces expressed the largest levels of genes encoding the related enzymes converting amino acids to higher alcohols, revealing that Zygosaccharomyces had a stronger ability to utilize amino acids and produce flavor compounds during Jiang-flavor *Baijiu* production.

In conclusion, this study provides a comprehensive analysis of the relationship between amino acids and microbial community assembly as well as their metabolic function during *Baijiu* fermentation. The information will help us to understand the microbial ecology mechanism and design strategies to improve food quality by manipulating microbial communities and their functioning. Further investigations are needed to elucidate the amino acid preferences of a larger collection of yeasts under culture-dependent conditions.

## MATERIALS AND METHODS

### Sample collection.

The experiment was conducted in Guizhou Guotai Liquor Group Co. Ltd., Guizhou Province, China (27°86′N, 106°38′E). Four fermentation batches with different amino acid contents were selected. Two batches had lower amino acid contents (group A) and another two batches had higher amino acid contents (group B). Samples were collected from two stages following a previously described method (20). Heap fermentation samples were collected daily (H1, H2, and H3). Pit fermentation samples were collected on days 0, 5, 10, 15, and 30 (F00, F5, F10, F15, and F30). Samples taken from different points in the same layer were mixed to form a sample to reduce the heterogeneity of samples (Fig. S1). Four biological repeats were adopted at every time point in each group. Finally, a total of 24 heap and 40 pit fermentation samples were obtained and used for further analysis.

### Quantitative analysis of carbohydrates and amino acids.

Carbohydrates were detected by Thermo ICS 5000+ Ion chromatography System (Thermo Fisher Scientific, Inc., Waltham. MA) equipped with Dionex CarboPac PA10 (4 × 250 mm) column and a pulsed amperometric detector. Samples were processed and detected according to the methods in previous studies (20, 54).

Seventeen free amino acids were determined. Samples (1 g) were homogenized with 5% trichloroacetic acid and diluted to 25 mL and then ultrasonically treated for 30 min and maintained for 2 h. The suspension was filtered by double-layer filter paper and centrifuged at 15,000 × *g* for 30 min, and then the supernatant was filtered using a 0.22-μm membrane filter and applied to an automatic amino acid analyzer (Agilent 1100 Series; Palo Alto, CA) equipped with Agilent Hypersil ODS column (5 μm, 4.0 mm × 250 mm), according to the method by a previous study (55). Each amino acid was identified and quantified by the retention time and peak area from the instrument software in comparison to FAA standards (Sigma Chemical Co., St. Louis, MO).

### Fermentation parameters detection and analysis.

Ethanol, lactic acid, and acetic acid in fermented grains were determined by high-performance liquid chromatography (HPLC 2695; Waters Corporation, Milford, MA) equipped with a refractive index detector (RI, 2414) at 35°C and an Aminex HPX-87H Ion Exclusion column (Bio-Rad, Hercules, CA), based on the methods in previous studies (20, 56, 57). The pH was measured with a pH meter (Delta320, Mettler Toledo, Switzerland) inserted directly into the sample site (10 g/100 mL). The temperatures of the sampling locations were measured using a digital stainless steel probe thermometer (T-80; Toolwell) and recorded before sample collection.

### Volatile metabolites analysis.

Volatile metabolites were determined by a headspace solid-phase microextraction combined with gas chromatography-mass spectrometry (HS-SPME-GC-MS) (GC 6890N and MS 5975; Agilent Technologies, Santa Clara) on a DB-Wax column (30 m × 0.25 mm inner diameter, 0.25-μm film thickness; J&W Scientific, Folsom, CA), based on the methods described in previous studies (58, 59).

### Total DNA extraction, amplification, and sequencing.

Total DNA was extracted by the EZN (Easy Nucleic Acid Isolation) soil DNA kit (Omega Bio-tek, Norcross, GA) following the instructions. For bacteria, the V3 to V4 hypervariable region of the 16S rRNA gene was amplified using the universal primer set 338F and barcode 806R (60). For fungi, the ITS region was amplified with the primers ITS1F and ITS2R (61). PCR products were purified using PCR purification kit, and the concentrations were determined by Thermo Scientific NanoDrop 8000 UV-Vis Spectrophotometer (NanoDrop Technologies, Wilmington, DE). The barcoded PCR products were sequenced using a MiSeq benchtop sequencer to obtain 250-bp paired-end reads (2 × 250 bp; Illumina, San Diego, CA) at Beijing Auwigene Tech. Ltd. (Beijing, China).

The generated raw sequences were performed using QIIME (V.1.9.1) (62). High-quality sequences were obtained by removing primer sequences, homopolymers <10, average quality scores <20, and the minimum length (excluding the primer or barcode region) <50 bp. Chimeric sequences were removed via USEARCH (V. 10.0.240) using the UCHIME algorithm (63). The OTUs of bacteria and fungi were clustered with a similarity threshold of 97% using Uclust (V. 1.2.22) (64). The representative bacterial OTU sequences were mapped to the Silva database (Release 138; http://www.arb-silva.de). The representative fungal OTU sequences were mapped to UNITE fungal ITS database (Release 8.2; http://unite.ut.ee/index.php) (65, 66). The OTUs were mapped to taxonomy using the RDP classifier of the QIIME pipeline. The data in each sample were treated homogeneously, and the subsequent diversity analysis was obtained based on the homogenized data. Shannon index was estimated by QIIME software (67).

### Total RNA extraction and metatranscriptomic sequencing.

All samples were treated with sterile phosphate-buffered saline (0.1 mol/L) and then centrifuged at 300 × *g* for 10 min. After filtering with gauze, the supernatant was then centrifuged at 10,000 × *g* for 10 min. The precipitated cells were milled with liquid nitrogen, and total RNA was extracted with sodium laurate buffer (10 g/L sodium laurate, 0.1 mol/L Tris-HCl, and 0.1 mol/L NaCl, and 0.02 mol/L EDTA) containing chloroform: isoamyl alcohol (24:1, vol/vol). The kit (Ribo-Zero; Epicentre, San Diego, CA) was used to remove the rRNA from the total RNA. Metatranscriptomic libraries were constructed according to the NEBNext Ultra RNA Library Prep kit (Illumina; New England Biolabs, Ipswich, MA) and were then sequenced on an Illumina HiSeqTM2500/4000 platform at Beijing Auwigene Tech. Ltd. (Beijing, China).

For annotation, similarity searches were performed to annotate unigenes against different databases using BLASTX. Kyoto Encyclopedia of Genes and Genomes (KEGG) analyses were performed. To understand the gene expression patterns in the different groups, fragments per kilobase of transcript per million mapped reads (FPKMs) were calculated for each sample, and all the unigenes were annotated. Differential miRNA expression analysis of the two libraries was performed using the DEGseq R package based on read count (68). The significant differentially expressed genes were evaluated against a |log_2_ (fold change)| of ≥2 and a false-discovery rate (FDR) of <0.1.

### Effect of serine on the growth and fermentation performance of Zygosaccharomyces bailii.

Z. bailii was isolated and identified according to the method described in our previous study (20). First, Z. bailii was precultured in 100 mL YPD medium (10 g/L yeast extract, 20 g/L peptone, and 20 g/L glucose) at 30°C and 200 rpm for 24 h to obtain 1.0 × 10^8^ cells/mL of seed culture. Then seed broth was centrifuged at 10,000 × *g* at 4°C for 5 min and the supernatant was discarded, and the resulting pellet was washed twice with sterile deionized water. Cells were thereafter resuspended in 100 mL of yeast nitrogen base (YNB) without amino acids (supplemented with 20 g/L glucose) and incubated under the above-mentioned conditions for 4 h to eliminate all intracellular nitrogen reserves. We used a synthetic medium containing carbohydrates according to their contents detected in fermented grains (20 g/L glucose, 1.0 g/L maltose, 0.5 g/L galactose, 1.5 g/L arabinose, and 0.1 g/L fructose) (Fig. S6) and 6.7 g/L YNB. The pH of the medium was adjusted to 3.70 by lactic acid. Subsequently, 100 mL of synthetic medium was poured into 250-mL conical flasks and supplemented with 100 mg/L and 300 mg/L serine, as well as 100 mg/L and 200 mg/L methionine, based on the contents in two groups of *Baijiu* fermentation (Table S1). A Synthetic medium without supplementation of amino acids was used as a control. Finally, 1 mL of precultures corresponding to an initial cell density of 1.0 × 10^6^ cells/mL was introduced to each treatment and cultured at 30°C and 200 rpm for 84 h. Every treatment was performed in triplicate. Samples were taken every 12 h to determine OD_600_, ethanol, and SAM concentrations.

Samples were centrifuged at 4°C and 8,000 × *g* for 5 min, and the supernatant was used to detection of ethanol according to the previously described method.

For SAM detection, 2 mL cell suspension was centrifuged at 8,000 × *g* for 5 min and cells were resuspended in 10 mL of 10% perchloric acid at 25°C for 2 h. Then the supernatant was filtered using a 0.22-μm membrane filter and applied to detect. SAM was detected by Waters HPLC 2695 system (Waters Corporation, Milford, MA) equipped with a UV detector and an Acquity HSS T3 analytical column (100 mm × 2.1 mm inner diameter, 1.8-μm film thickness; Waters, Milford, MA) with a mobile phase containing 50 mM KH_2_PO_4_ and 5% methanol adjusted to pH 2.5 with H_3_PO_4_. The wavelength used for UV detection was 210 nm.

The effect of SAM on the growth of Z. bailii was carried out using the above-mentioned synthetic medium by supplementing 30 mg/L SAM.

Transcription analysis of genes from serine to SAM was performed by reverse transcription-quantitative PCR (RT-qPCR) according to the method in our previous study (56). The primers for RT-qPCR for the genes in Z. bailii were listed in Table S2.

### Ecological community assembly analysis.

The principle to quantify the community assembly process is based on phylogenetic βNTI. We compared the βNTI of the microbial community within one sampling time pairwise, as described by Stegen et al. (69). |βNTI| > 2 reveals that the deterministic process governs the observed turnover between pairwise communities. In detail, βNTI ≤ 2 indicates homogeneous selection, and βNTI > 2 indicates variable selection. In contrast, |βNTI| < 2 indicates that the stochastic process plays an important role.

### Statistical analyses.

Statistical analyses and the generation of plots were performed using OriginPro2020, Microsoft Excel, and Adobe Illustrator CC2018. Statistical differences were obtained using a one-way ANOVA followed by Turkey’s test. An adjusted *P* value of <0.05 (with false discovery rate correction) was used as the significance threshold. PCoA was conducted to calculate the differences of microbial community between two groups by Canoco software. Volatile compounds (Z-score transformed) were visualized by heatmap constructed using R (v.3.6.1) via the pheatmap package (v.1.0.12). VPA was used to determine the relative importance of amino acids, carbohydrates, fermentation parameters, and interactions between these factors to the microbial community composition and was performed by the “varpart” function in Vegan package (V. 2.4-3) in R (70). To evaluate the correlations between the microbial community and various parameters, RDA was conducted using the function “capscale” in vegan package (V. 2.4-3) in R. NMDS was conducted using R. To evaluate the relationships among different microorganisms in each group, cooccurrence network analysis was conducted. All possible Spearman’s rank correlations between the abundant genera (average abundance >0.1%) in each group were calculated using R (v.3.6.1) via psych and reshape2 packages (71). Only significant (*P < *0.05, with false discovery rate correction and |ρ| > 0.6) correlations were considered valid correlations. Networks were constructed using Gephi (Web Atlas, Paris, France) to visualize the correlations (72). Major network topological indices were calculated in the Gephi to characterize the topological structure of networks, including modularity, average degree, positive, and negative portions. Similarly, Spearman correlations between amino acids and yeasts were calculated and visualized. All the data are expressed as means ± standard deviation.

### Data availability.

The fungal and bacterial raw sequence data were deposited in the NCBI SRA and are available under accession numbers PRJNA744402 and PRJNA744627. The metatranscriptomic data were submitted to NCBI SRA and are available under accession numbers PRJNA744944.
