# Supplementary material for: Amino Acids Drive the Deterministic Assembly Process of Fungal Community and Affect the Flavor Metabolites in Baijiu Fermentation
Source: Microbiol Spectr. 2023 Mar 21;11(2):e02640-22. doi: 10.1128/spectrum.02640-22 (PMC10100711; doi:10.1128/spectrum.02640-22)
Supplement: Supplemental file 1 — Supplemental material. Download spectrum.02640-22-s0001.pdf, PDF file, 1.3 MB [file spectrum.02640-22-s0001.pdf]

Supplemental materials

Amino acids drive the deterministic assembly process of fungal community and affect the flavor metabolites in *Baijiu* fermentation

Junlin Wei[a], Jun Lu[b], Yao Nie[a], Changwen Li[b], Hai Du[a]*, Yan Xu[a]

[a]Laboratory of Brewing Microbiology and Applied Enzymology, Key Laboratory of Industrial Biotechnology of Ministry of Education, School of Biotechnology, Jiangnan University, 1800 Lihu Avenue, Wuxi, Jiangsu 214122, China

[b]Guizhou Guotai Liquor Group Co. Ltd, Guizhou 564501, China

* Corresponding author:

Dr. Hai Du

Jiangnan University, Wuxi 214122, China

Phone: +86-510-8591-8201

E-mail: **duhai88@126.com**

Supplemental Figures

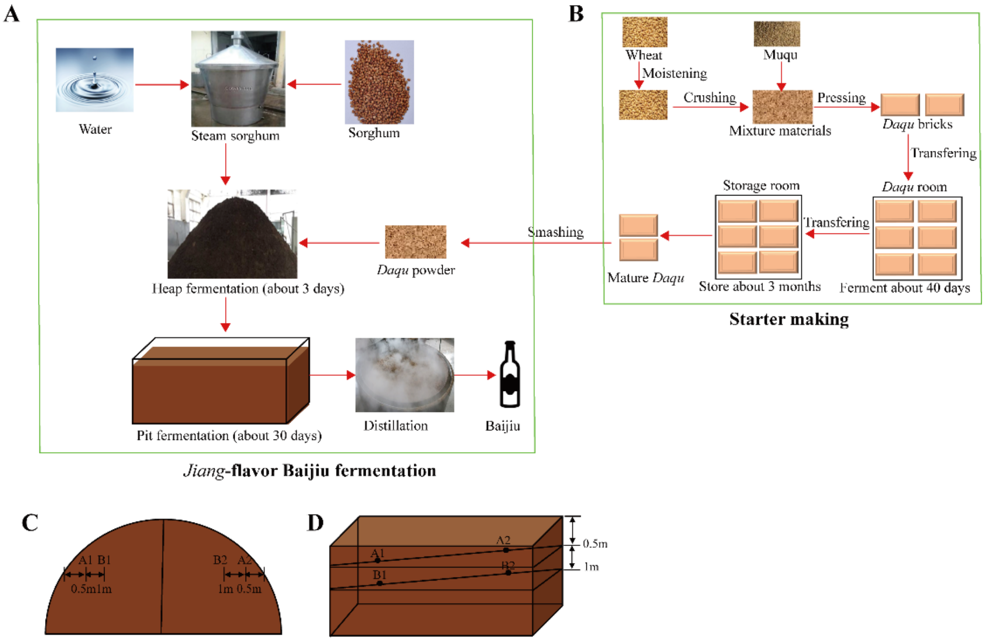

Fig. S1 Production process of Jiang-flavor *Baijiu*. (A) The complete production process of *Baijiu*. (B) The complete production process of *Daqu* (starter). (C) Schematic diagram of the sampling method during heap fermentation. “A1, A2” and “B1, B2” samples were taken from the same depth but at different locations. A final sample was made by mixing the samples from the same depth (A1 and A2, B1 and B2) to reduce the volatility of samples. (D) The schematic diagram of sampling during pit fermentation. “A1, A2” and “B1, B2” indicated that samples were taken from the same depth but at different locations. A final sample was made by mixing samples from the same depth (A1 and A2, B1 and B2) to reduce sample volatility.

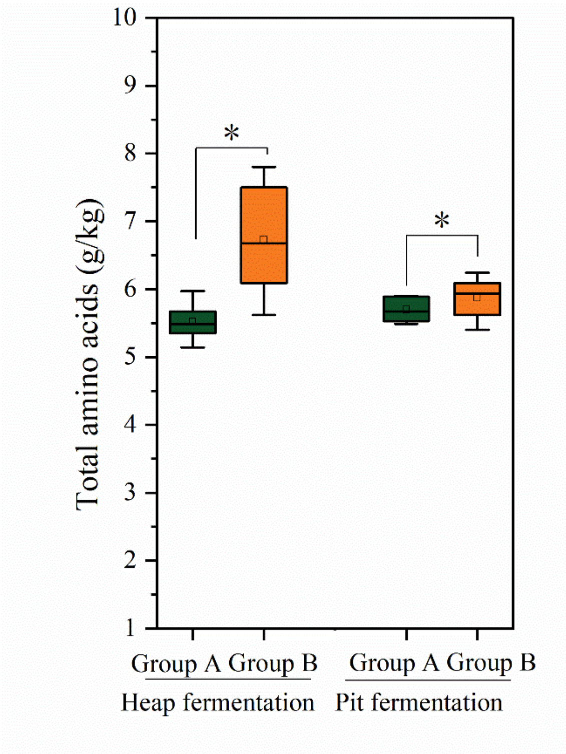

Fig. S2 Comparison of total amino acids during heap and pit fermentation in group A and group B. *, adjusted $P < 0.05$ (Tukey's test).

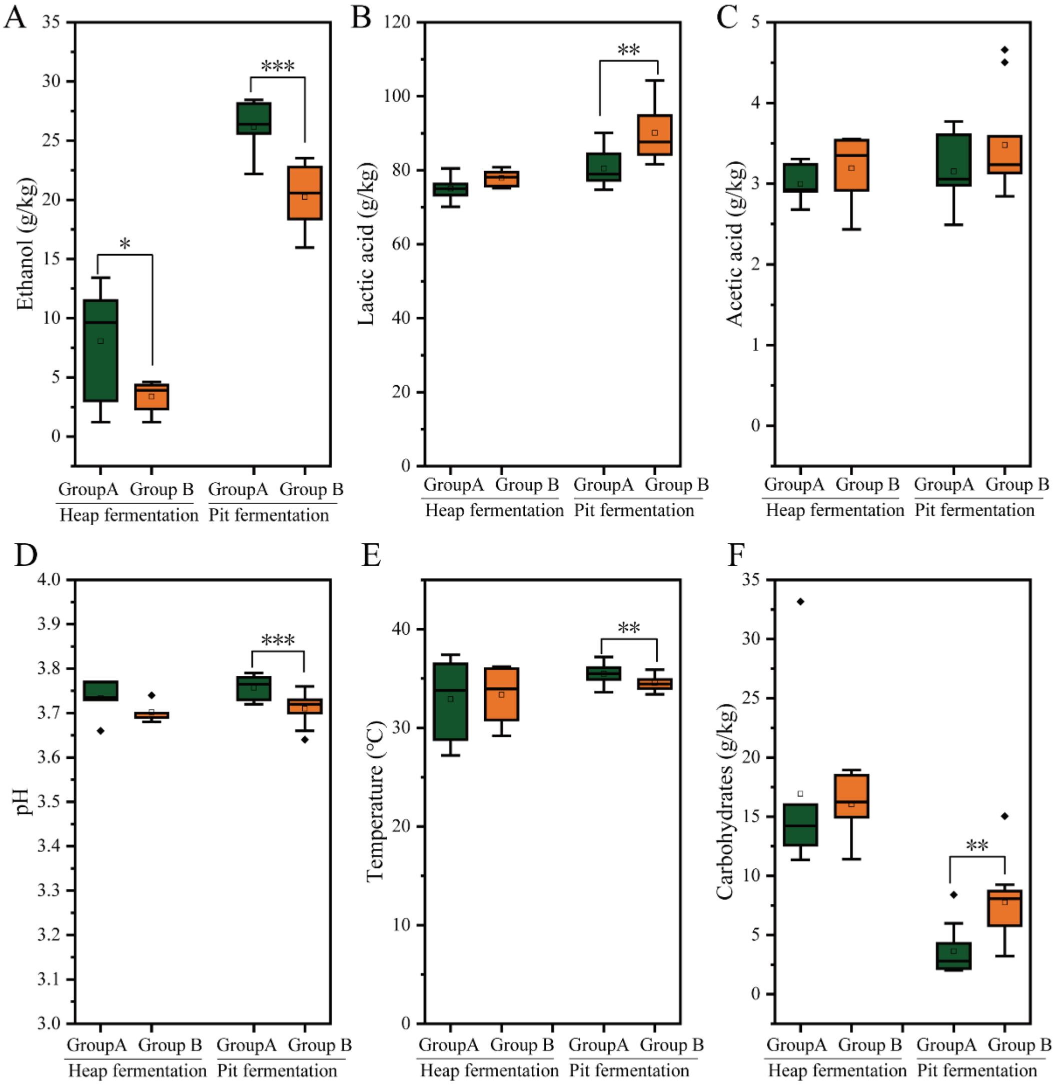

Fig. S3 Comparison of fermentation parameters during heap and pit fermentation in group A and group B. **, adjusted $P < 0.01$; ***, adjusted $P < 0.001$ (Tukey's test).

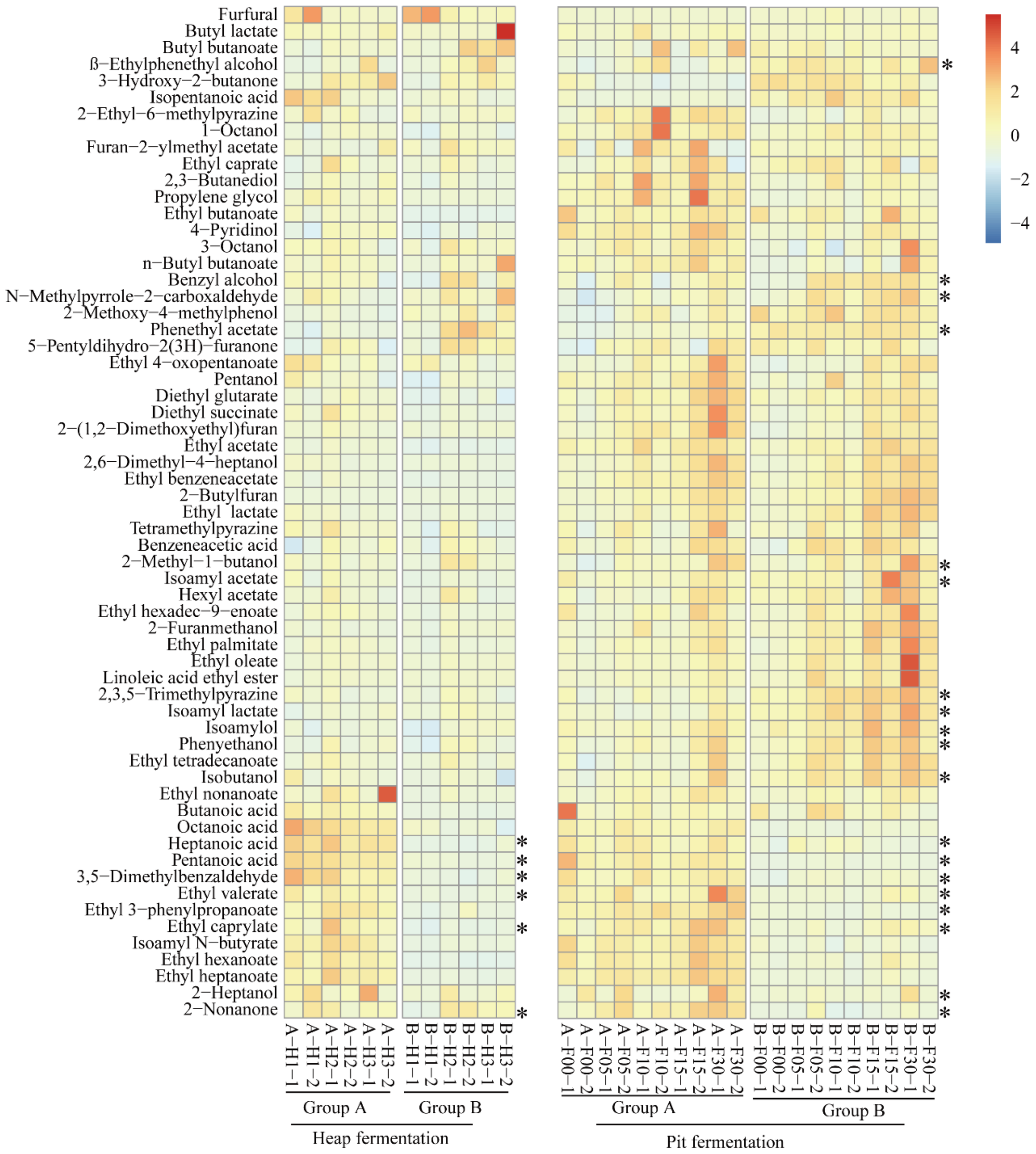

Fig. S4 Comparison of volatile metabolites during heap and pit fermentation in group A and group B. The color scale represents the scaled abundance of each metabolite, indicated as the Z-score, with red and blue indicating high and low abundances, respectively. H represents heap fermentation and F represents pit fermentation. The numbers after the letters represent the number of fermentation days. "-1" represents one batch in a group, and was averaged by two sample points (see sample collection); "-2" represents another batch in a group. *, adjusted $P < 0.01$ (Tukey's test).

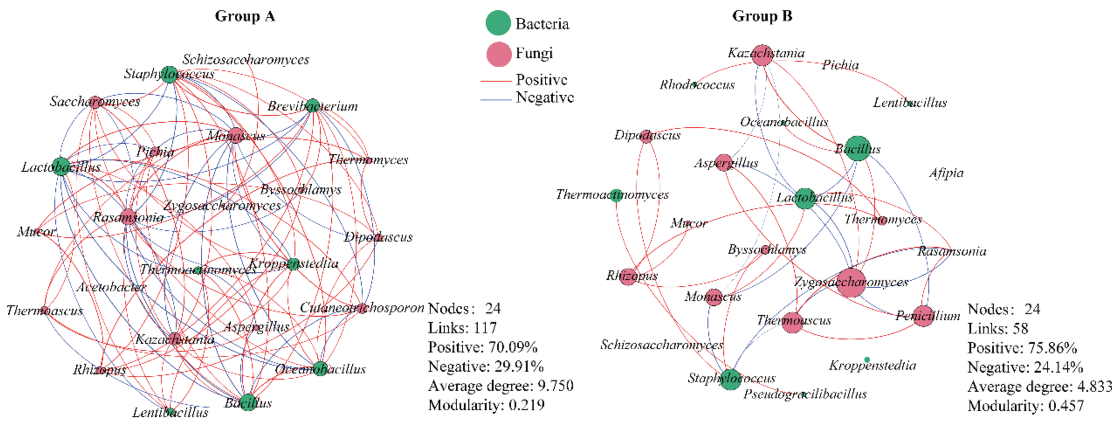

Fig. S5 Co-occurrence networks of the microbial communities in two groups. A connection indicates a significant (adjusted $P < 0.05$) and strong (Spearman's $|\rho| > 0.6$) correlation. The thickness of each connection (edge) between two nodes is proportional to Spearman's correlation coefficient (ρ). The colour of each edge corresponds to a positive (red) or negative (blue) relationship.

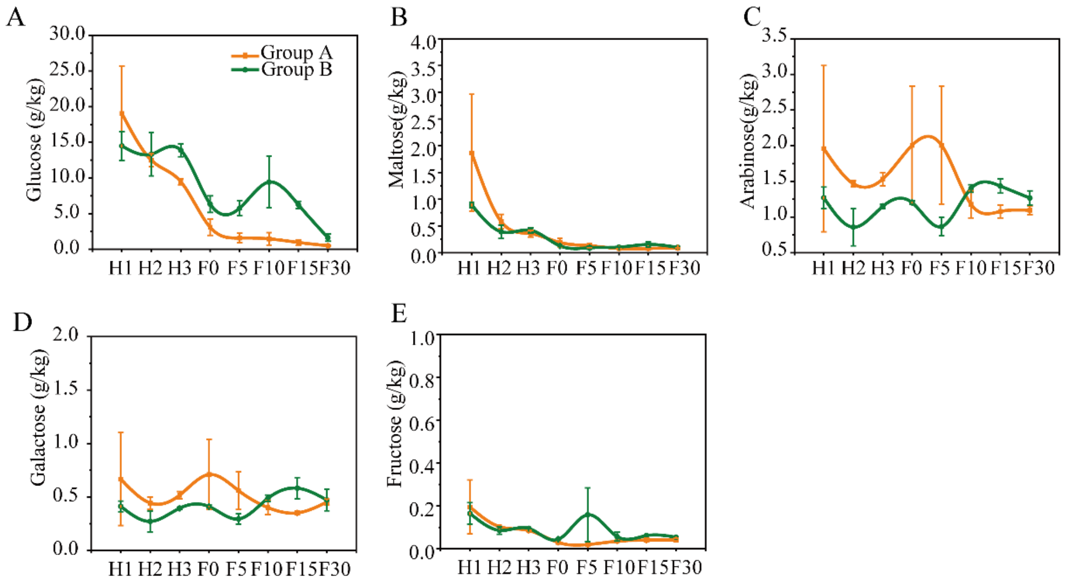

Fig. S6 Carbohydrate profiles during *Baijiu* fermentation in two groups. (A) Glucose; (B) Maltose; (C) Arabinose; (D) Galactose; (E) Fructose. H represents heap fermentation and F represents pit fermentation. The numbers after the letters represent the number of fermentation days. Each value represents the average and error bars represent the standard deviation (SD) (n=4). *, adjusted $P < 0.05$ (Tukey's test).

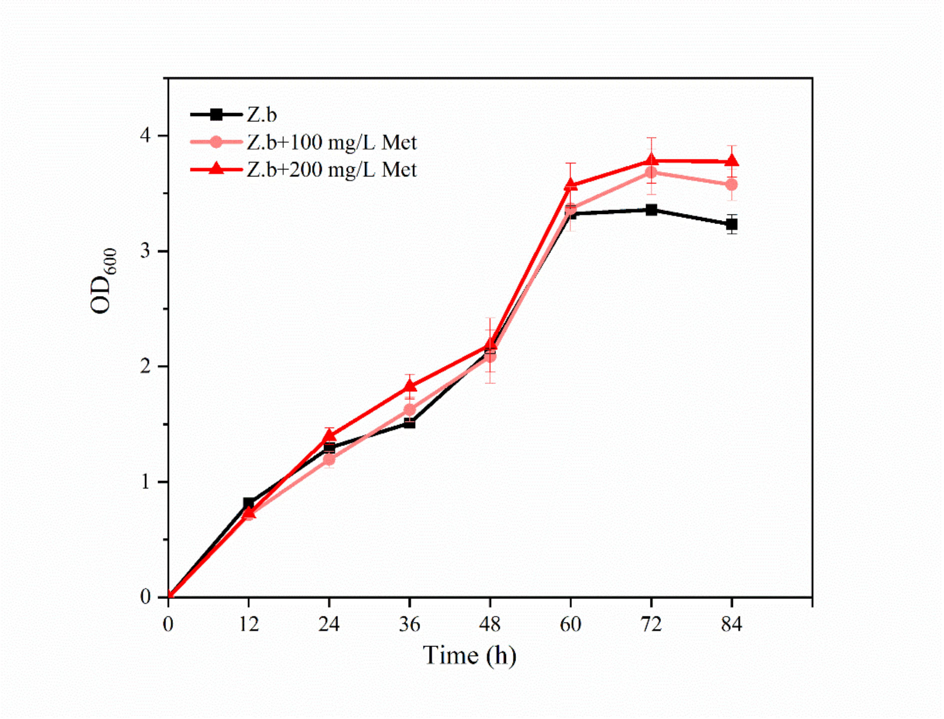

Fig. S7 OD_{600} values of *Z. bailii* during fermentation in the presence of 100 mg/L and 200 mg/L methionine (n = 3). Z.b: *Z. bailii*; Met: Methionine.

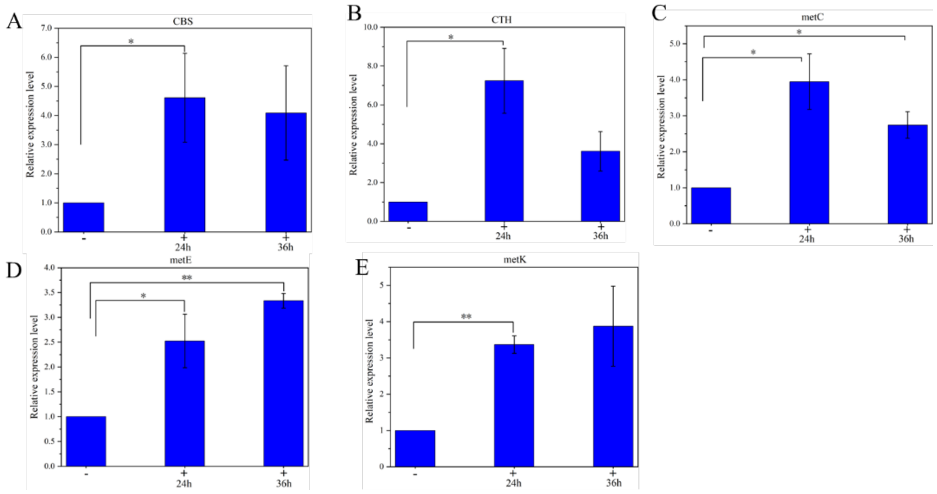

Fig. S8 Relative transcription ratios of genes associated with S-Adenosyl-L-methionine production from serine in *Z. bailii* with the presence of 300 mg/L serine (+) as compared to the control (-: no serine). The expression level in the control group was regarded as 1. Each value represents the average and error bars represent the standard deviation (n = 3). *, adjusted $P < 0.05$; **, adjusted $P < 0.01$ (Tukey's test).

CBS: cystathionine beta-synthase; CTH: cystathionine gamma-lyase; metC: cysteine-S-conjugate beta-lyase; metE: 5-methyltetrahydropteroyltriglutamate--homocysteine methyltransferase; metK: S-adenosylmethionine synthetase.

Table S1 Dynamics of each amino acid in two groups during *Baijiu* fermentation. H represents heap fermentation and F represents pit fermentation. The numbers after the letters represent the number of fermentation days. All data are presented as mean ± standard deviation.

**Amino acid (mg/kg)**	**A-H1**	**A-H2**	**A-H3**	**A-F00**	**A-F05**	**A-F10**	**A-F15**	**A-F30**	**B-H1**	**B-H2**	**B-H3**	**B-F00**	**B-F05**	**B-F10**	**B-F15**	**B-F30**
Aspartic acid	305.77±1.51	257.66±12.60	325.16±30.50	336.51±28.43	316.51±23.46	326.43±28.78	318.00±28.90	330.04±3.10	310.75±16.91	317.52±18.40	273.47±3.79	305.00±8.00	267.70±6.78	245.87±4.35	250.23±50.98	257.68±63.76
Glutamic acid	572.47±16.76	561.70±28.15	638.91±49.48	649.11±24.57	649.11±19.89	635.01±18.99	698.91±44.56	640.03±5.11	593.73±2.84	617.01±27.41	552.11±73.11	645.74±1.33	635.44±3.67	652.67±45.67	648.34±50.42	658.79±60.62
Serine	90.01±9.46	258.94±60.45	370.50±114.12	349.13±37.01	323.13±25.04	221.54±23.98	270.56±14.12	255.64±9.25	435.18±22.75	352.69±36.93	324.64±34.04	379.61±25.09	367.81±15.29	354.64±30.09	360.12±30.98	366.06±41.10
Histidine	118.58±13.58	145.59±11.45	119.19±4.41	134.56±30.43	143.56±29.89	145.09±26.78	121.09±6.78	138.47±27.59	343.37±26.92	141.63±22.78	141.06±34.63	137.89±14.62	141.19±9.89	135.06±30.90	140.45±4.09	141.42±3.66
Glycine	272.53±11.83	280.74±10.80	296.23±39.43	314.48±61.40	309.48±56.78	299.88±49.09	289.03±34.43	293.32±15.16	418.82±24.78	330.39±8.67	303.74±33.60	327.26±21.24	309.13±18.79	298.74±23.60	309.34±10.98	338.68±14.35
Threonine	290.15±7.78	237.69±15.75	221.66±7.90	229.96±47.67	231.76±37.67	254.56±38.09	219.96±17.90	228.42±19.07	438.39±28.39	438.36±29.22	328.11±38.41	243.85±18.55	223.56±12.34	228.09±28.97	243.15±7.87	246.85±6.78
Arginine	347.14±36.68	351.56±27.00	346.24±35.47	393.73±28.52	409.67±22.21	476.43±21.09	436.24±28.79	444.69±4.38	366.14±8.31	346.94±8.71	350.58±59.86	391.65±0.95	400.15±9.80	450.08±40.09	409.51±13.45	438.41±14.65
Alanine	584.99±18.71	596.09±31.82	637.57±68.64	651.05±61.67	656.45±55.89	632.34±45.67	667.34±67.64	639.20±4.03	671.39±14.53	654.35±14.31	630.06±21.33	666.34±18.45	623.78±20.98	620.90±20.09	638.54±40.89	688.43±36.33
Tyrosine	274.20±13.95	296.38±13.21	347.21±48.74	321.73±22.04	309.21±21.90	321.21±19.08	313.21±45.74	345.07±4.30	435.52±18.82	313.15±9.92	309.35±30.44	326.52±5.62	310.52±9.09	323.35±30.09	324.07±8.09	348.57±7.75
Cysteine	131.08±8.23	29.36±25.41	45.25±36.11	82.50±9.49	97.32±7.98	88.12±8.09	49.25±45.66	58.41±23.84	80.51±5.10	14.81±4.45	74.05±22.02	26.83±21.61	29.90±8.90	30.05±12.02	48.19±20.98	51.89±36.81
Valine	286.07±10.63	283.09±20.70	335.90±44.09	334.70±33.74	323.40±22.56	321.54±19.77	321.90±35.60	336.49±10.05	578.65±5.97	327.84±48.75	341.90±23.43	377.92±5.67	366.56±4.67	346.56±20.09	332.56±4.78	354.38±53.08
Methionine	124.46±38.54	135.03±1.49	147.05±0.31	141.95±8.84	146.56±8.98	135.56±9.78	140.05±1.67	146.58±6.72	159.64±9.21	177.96±0.74	154.50±8.89	176.25±11.82	156.65±9.08	167.06±4.56	163.78±6.78	173.04±1.58
Phenylalanine	453.49±204.05	413.75±41.55	449.20±129.84	361.19±0.87	376.23±0.23	345.32±5.89	349.54±34.56	398.04±128.32	660.76±125.79	627.62±72.27	536.28±44.25	514.26±57.26	423.45±23.46	436.56±34.89	400.32±11.89	389.88±23.56
Isoleucine	241.43±1.31	258.00±7.77	269.04±4.22	257.85±22.25	247.56±21.90	234.56±19.88	245.04±3.59	276.09±0.70	483.90±18.50	427.63±11.75	367.66±37.12	280.55±1.91	234.55±2.34	267.56±20.98	282.89±4.56	292.97±3.24
Leucine	495.52±21.88	487.86±28.34	521.37±67.09	542.89±43.12	535.45±33.12	576.55±35.77	567.37±55.67	601.34±7.87	695.21±27.35	539.17±13.32	520.86±48.38	554.00±5.16	467.06±6.78	560.16±28.38	600.18±10.98	624.28±15.79
Lysine	257.03±6.51	130.86±0.63	140.06±16.96	147.36±14.90	134.56±15.40	145.54±16.57	146.09±17.11	145.07±4.72	421.87±2.19	395.62±2.24	273.06±2.09	132.94±1.13	114.04±2.45	173.12±12.09	172.09±10.98	122.14±13.53
Proline	704.93±46.62	512.19±8.17	509.03±30.71	506.66±23.12	521.33±22.56	508.55±22.09	523.09±20.71	510.65±8.47	703.11±11.29	653.96±4.82	439.13±42.00	481.46±62.67	523.42±54.98	533.23±23.45	546.03±9.09	596.33±10.23

Table S2 Primer sequences for gene transcription analysis in *Z. bailii*.

gene	primer direction	Primer sequence (5'→3')	primer size(bp)
CBS	F	CCACTGTGCAAGATTTGAAG	20
	R	GACAAAGTAACTAGTCCGCT	20
CTH	F	TCGCCTTATTTGTCTAACCC	20
	R	GTGGACCAACCAACTATCAA	20
metC	F	GCATTGGACACGATTATACG	20
	R	GAGGAACGTAAGCAACCTAT	20
metE	F	TTCCAAATCGTCAACCTCAT	20
	R	GTCTGTCAAGGAATCTGTGT	20
metK	F	GTCGTAACCGATCTTCTTGA	20
	R	AGATCTGTGACCAAGTTTCC	20
UBC6	F	GCAGACGATCAATGGAGTAT	20
	R	GCAGACGATCAATGGAGTAT	20
